# Recombinant production of a functional SARS-CoV-2 spike receptor binding domain in the green algae *Chlamydomonas reinhardtii*

**DOI:** 10.1371/journal.pone.0257089

**Published:** 2021-11-18

**Authors:** Anthony J. Berndt, Tressa N. Smalley, Bijie Ren, Ryan Simkovsky, Amr Badary, Ashley E. Sproles, Francis J. Fields, Yasin Torres-Tiji, Vanessa Heredia, Stephen P. Mayfield

**Affiliations:** Division of Biological Sciences, University of California San Diego, La Jolla, California, United States of America; Arizona State University, UNITED STATES

## Abstract

Recombinant production of viral proteins can be used to produce vaccine antigens or reagents to identify antibodies in patient serum. Minimally, these proteins must be correctly folded and have appropriate post-translation modifications. Here we report the production of the SARS-CoV-2 spike protein Receptor Binding Domain (RBD) in the green algae *Chlamydomonas*. RBD fused to a fluorescent reporter protein accumulates as an intact protein when targeted for ER-Golgi retention or secreted from the cell, while a chloroplast localized version is truncated. The ER-retained RBD fusion protein was able to bind the human ACE2 receptor, the host target of SARS-CoV-2, and was specifically out-competed by mammalian cell-produced recombinant RBD, suggesting that the algae produced proteins are sufficiently post-translationally modified to act as authentic SARS-CoV-2 antigens. Because algae can be grown at large scale very inexpensively, this recombinant protein may be a low cost alternative to other expression platforms.

## Introduction

In late 2019 a novel coronavirus was identified in the Wuhan province of China with a genome sequence closely resembling that of the Severe Acute Respiratory Syndrome (SARS) coronavirus identified in 2003. Thus, the novel coronavirus was named SARS-CoV-2. The respiratory disease the virus causes has since been named COVID-19 (COronaVIrus Disease 2019). The virus quickly spread worldwide and was classified as a global pandemic in April of 2020 [1], and has continued to spread more than a year after its initial identification.

Widespread use of nucleic acid tests that detect the SARS-CoV-2 RNA genome, such as RT-qPCR, have become the standard method to detect viral infection. However, laboratory assays that measure antibody responses and determine seroconversion are not yet comparatively as widely available. While such serological assays are not well suited to detect acute infections, and indeed antibody production lags days behind symptoms and infectiousness, multiple relevant applications exist for such antibody tests as they are one of the best indicators of prior immune responses to viral antigens, and therefore might indicate individuals are immune [2]. In addition to use as a reagent to identify serum antibodies, recombinant spike protein can also function as a vaccine antigen, and many of the SARS-CoV-2 vaccines in development or currently approved are based on the SARS-CoV-2 spike protein [3]. As these vaccines are deployed at a population scale, monitoring short- and long-term immune response to the vaccine target (the SARS-CoV-2 spike protein) will be a key component of characterizing vaccine efficacy, but will also potentially provide information of when booster-doses of the vaccines might be required, as immune responses can wane over time, while the virus is still known to be circulating in the population. This may become especially important as variable adherence to vaccination schedules established during clinical trials and “mix and match” use of different vaccines becomes a practical reality. To develop such antibody tests, it is critical to produce the viral protein antigens at a large scale and at an affordable price, so that antibody tests can become available around the world, and not just in the economically advantaged countries.

The spike protein of SARS-CoV-2 mediates viral entry into host cells by first binding to the host angiotensin-converting enzyme 2 (ACE2) receptor followed by fusion of the viral and host membranes and release of the viral RNA into the host cell [4, 5]. The receptor-binding domain (RBD) of the spike protein is located in the S1 subunit and directly mediates the interaction with the ACE2 cell receptors [4]. Patients that have recovered from SARS-CoV-2 infection usually have serum antibodies directed against the SARS-CoV-2 RBD [6, 7]. High levels of serum RBD specific binding antibodies in convalescent patient serum correlate well with viral neutralization and many neutralizing antibody clones isolated from convalescent patients cluster around the receptor binding domain of the spike protein [7–10]. Based on these findings, the SARS-CoV-2 RBD has become a common antigen used for serological assays and represents a common antigenic target of multiple vaccines [3].

Production of useful SARS virus spike proteins and their subdomains has been a challenge for non-animal cell production systems. Production of the 2003 SARS virus RBD or full spike protein utilizing an *E*. *coli*-based system, showed poor protein folding and aggregation into inclusion bodies. The resulting protein was much less immunogenic than the same protein produced in mammalian or insect cells [11, 12]. Production of coronavirus RBD proteins in fungal systems such as *Pichia pastoris* have demonstrated potential, but the productivity tends to be orders of magnitude lower than what these yeast systems have been shown to be capable of for other recombinant proteins [13, 14].

Previous studies have shown success in plant-based systems for recombinant production of viral recombinant proteins [15–18]. The membrane (M) and nucleocapsid (N) proteins of SARS were expressed transiently in *Nicotiana benthamiana*, with the N protein demonstrating antibody recognition in convalescent serum samples [18]. More recent work in *N*. *benthamiana* has demonstrated transient expression of both receptor binding and S1 domains of the SARS-CoV-2 spike protein and successful downstream application to immunoassays [19, 20]. The main drawback of plant-based expression systems is that they have low biomass productivity compared to microbial systems and have laborious and technically demanding transformation methods that can require months to achieve if stable integration is required [21].

Green microalgae can be grown photosynthetically or heterotrophically and can scale very rapidly [22]. Algae have been demonstrated to fold complex eukaryotic proteins [23, 24], to be amenable to sophisticated molecular genetic tools [25], and to express recombinant proteins which can be directed to any subcellular structures [26]. Collectively this allows for the rapid production of complex proteins that can be grown at large scale in a cost effective manner [27]. Recently, *N*. *benthamiana* agrobacterium-mediated transformation vectors have been used to transiently express SARS-CoV-2 RBD in both *Chlamydomonas reinhardtii* and *Chlorella vulgaris* [28]; however, evaluation of the functionality of the recombinant RBD at its cognate ACE2 receptor was not determined.

Here we examined the potential of utilizing nuclear transformation in the unicellular green microalgae *C*. *reinhardtii* as a production platform for recombinant SARS-CoV-2 spike RBD protein. We have produced stable nuclear genome integration vectors that generate an order of magnitude higher recombinant RBD expression levels compared to previously reported methods in *C*. *reinhardtii* and maintain this expression for at least one year after transformation. Three different strategies for recombinant protein production within *C*. *reinhardtii* were tested by appending different intracellular localization motifs to the transgene. The RBD proteins were targeted to be retained in the Endoplasmic Reticulum-Golgi pathway, secreted out of the cell into the culture media, or targeted for accumulation within the chloroplast. We found that recombinant RBD targeted to the chloroplast accumulated to high levels, but appeared to be truncated by ~5 kDa at the amine end of the mature protein, and this protein was not recognized by anti-RBD antibodies in Western Blotting assays. RBD proteins targeted to the ER or secreted from the cell produced a protein of the expected size and correct amino acid sequence. We purified spike RBD protein from the ER-Golgi retained version and demonstrated that it specifically binds to recombinant ACE2 protein at a similar affinity as mammalian expressed RBD. These data demonstrate the potential of using eukaryotic algae as an efficient and scalable platform to make correctly folded and functional SARS-CoV-2 RBD recombinant proteins that could be used in large scale antibody assays or as potential vaccine antigens.

## Results

### Design of SARS-CoV-2 spike protein RBD expression cassette for recombinant protein production in algae

**Fig 1:**
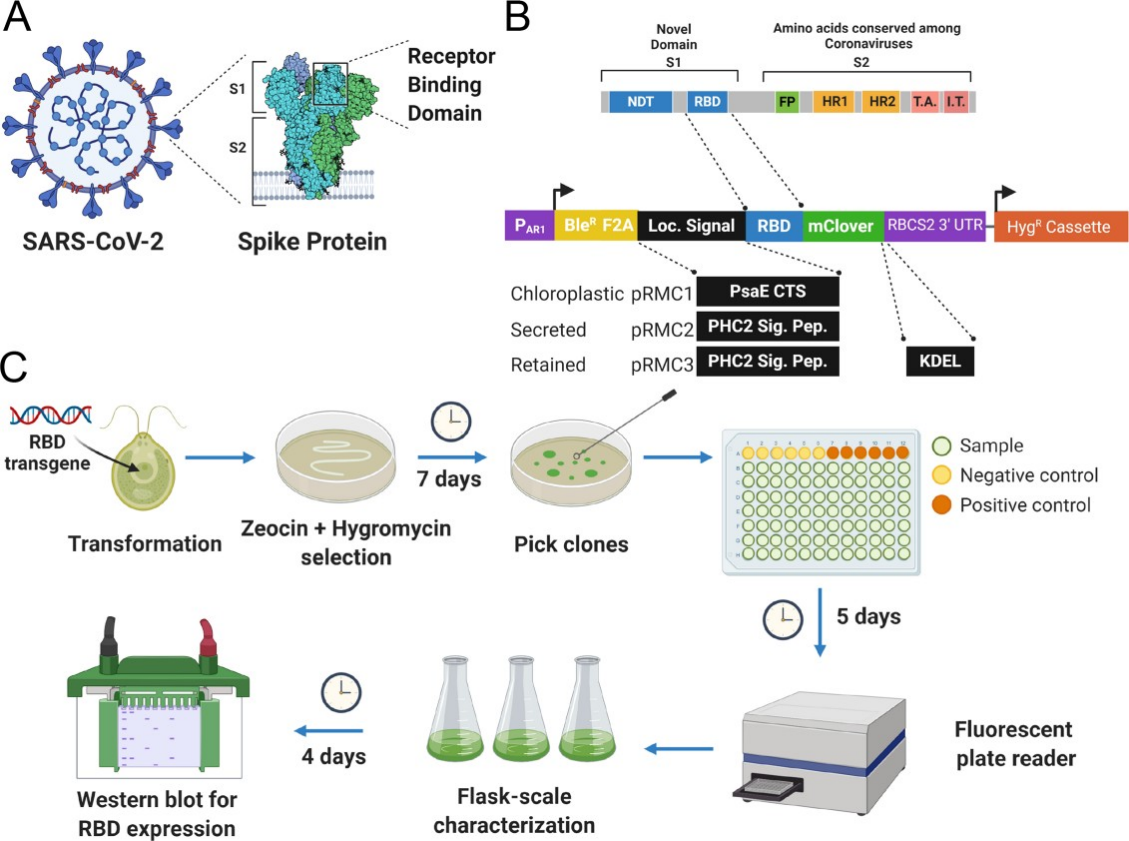
Summary of RBD::mClover expression vector design and algal transformant selection process. (A) Diagram of the SARS-CoV-2 viral particle with crystal structure of the spike (S) protein highlighted with Subunits 1 and 2 indicated (S1, S2, respectively). The Receptor Binding Domain (RBD) is located in the more variable **S**ubunit 1. (B) Vector design and construction. The peptide structure of the spike protein indicating the N-Terminal Domain (NTD), Receptor Binding Domain (RBD), Fusion Peptide (FP), Homology region 1 and 2 (HR1, HR2), Transmembrane association domain (TA) and Intracellular Terminal (IT). A *C*. *reinhardtii* nuclear codon optimized version of the RBD-coding sequence was cloned in to a vector containing the AR1 promoter (P_AR1_) driving a transcriptional fusion of the Bleomycin resistance gene (Ble^R^), Foot-and-mouth disease virus 2A (F2A) ribosomal-skip motif and 5’ mClover green fluorescent protein tag. A separate Beta-tubulin2 promoter driving Hygromycin resistance was used for secondary selection. Three different versions of the RBD were generated. A chloroplast-directed version through N-terminal fusion of the PsaE chloroplast transit sequence, a secreted version by the addition of the PHC2 secretion signal peptide, and an ER-Golgi system retained version by the subsequent addition of a C-terminal KDEL Golgi retention sequence. (C) Schematic summarizing transformation process and timeline including drug selection, clone down selection through 96-well microtiter plates, and then flask-scale characterization of candidate RBD-expressing lines.

Based on previous studies, as well as bioinformatics and structural modeling, we elected to produce amino acids 319–537 of the SARS-CoV-2 spike protein comprising the RBD [29] (Fig 1A). For high throughput screening to rapidly identify recombinant protein production, we fused this domain to the N-terminus of the green fluorescent protein (GFP) derivative mClover [30]. The *C*. *reinhardtii* nuclear genome codon optimized fusion genes of the spike-RBD and mClover were then placed in a modified expression vector based on components of the previously published pBR9 and pOpt vectors [26, 31] (Fig 1B). Expression was driven with the semi-synthetic AR1 promoter [23]. A 5’ Bleomycin resistance gene (*Ble*^*R*^) was included as part of the expression cistron to allow for selection of high expressing clones on Zeocin containing media, while a Foot-and-mouth disease virus 2A ribosomal-skip motif was placed between the *Ble*^*R*^ coding region and the RBD::mClover fusion protein, so that the RBD::mClover accumulated as a single fusion protein [23]. A separate Hygromycin resistance gene driven by a beta-tubulin promoter was placed 3’ of the RBD transgene to allow for double selection (Zeocin and Hygromycin), ensuring that the RBD::mClover transgene is intact in any transformant selected [32].

### Targeting of the RBD::mClover protein to subcellular compartments in transgenic algae

Since it is known that there are different protein folding and post-translational modifications made to proteins depending on which organelle a protein is localized, we generated three versions of the RBD::mClover construct; 1) A chloroplast directed version, produced by adding the psaE chloroplast transit sequence to the N-terminus of the RBD fusion protein, 2) a secreted version, produced by adding Pherophorin 2 (PHC2) signal peptide to the N-terminus of the RBD fusion protein, and 3) an ER-Gogli retained version produced by the addition of a C-terminal KDEL retention motif to the carboxy end of the RBD fusion protein containing the PHC2 secretion peptide (Fig 1B).

### Transformation and high throughput screening for recombinant protein production

The three vectors were linearized and transformed separately into algae via electroporation. Following overnight recovery in complete liquid media, cells were selected on media containing both Zeocin and Hygromycin. Ten days post-transformation, individual colonies were picked into 96-well microtiter plates containing TAP media and grown for two days. The clones were then passaged at a 1:4 dilution in to fresh TAP media for two days and fluorescence analysis, using a plate reader, was used to identify strains with high mClover expression (Fig 1C). The mClover fluorescence signal was normalized to chlorophyll fluorescence and compared to both the CC124 starting strain and a previously characterized GFP-expressing strain [33]. Several hundred mClover expressing transformants were recovered for both the secreted and ER retained constructs, from three independent transformations, while only a few dozen colonies were recovered for the chloroplast targeted construct, despite the same amount of DNA being used in each of the transformations. Similarly, by mClover fluorescence analysis, 10–30% of all secreted and ER retained transformants showed fluorescence well above wild type, while only about 1–5% of the chloroplast-directed strains showed any mClover fluorescence.

### Characterization of RBD::mClover localized to different compartments

**Fig 2:**
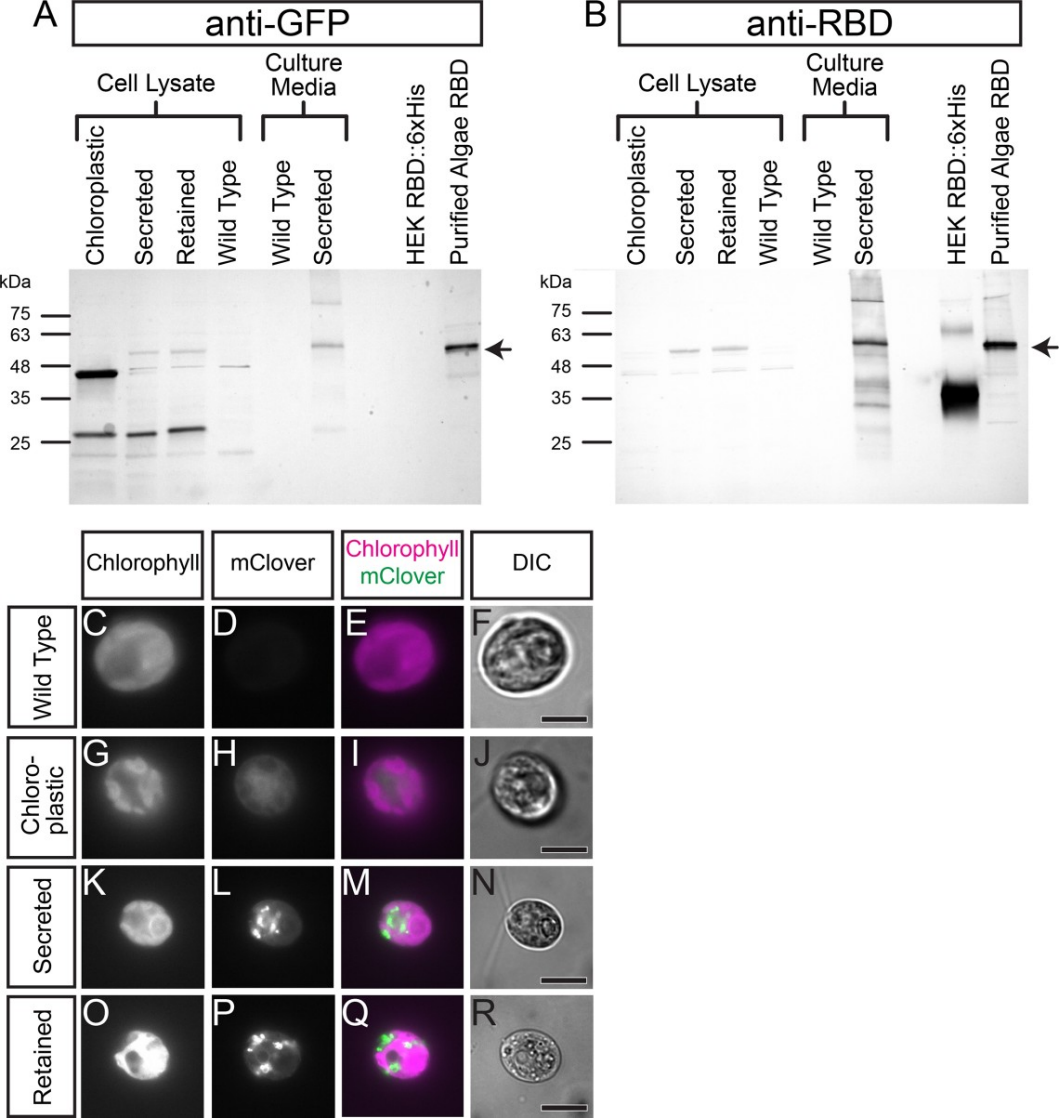
Characterization of RBD::mClover expression with different subcellular localizations. (A) Western Blot characterizing the expression of RBD::mClover in the cell pellet lysate or secreted supernatant of transformants from vectors pRMC1/2/3 by probing the blot with anti-GFP. 5 μg of total protein from the cell pellets were loaded in to each well while a 10 μL of 1:200 concentration of the cell culture supernatant or 5 μL of partially purified Algae RBD::mClover was loaded. 100 ng of HEK-cell produced RBD::6xHis tag was used as a control. A background band is observed at ~45 kDa in all samples including our wild-type control. The expected size of all RBD::mClover gene products is 51 kDa and is indicated by an arrow on the right-hand side of the blot. The gene product of the Chloroplast-directed version of our RBD construct appears as a strong band below the 48 kDa molecular weight marker while the Secreted or ER-Golgi retained versions appear above the 48 kDa marker. (B) A Western Blot with the same organization as before but probed with Rabbit anti-RBD polyclonal antibody. The Chloroplast-directed version of RBD::mClover does not appear to be detected while the Secreted and ER-Golgi retained versions are. A background band is observed in all samples including the wild type control at ~51 kDa. (C-R) Fluorescence and Differential Interference Contrast microscopy (DIC) images of wild type and RBD::mClover construct transformed *C*. *reinhardtii* cells. (G-J) The chloroplast-directed RBD::mClover shows little to no co-localization with the chlorophyll auto-fluorescence of the chloroplast. (K-R) The secreted and ER-Golgi retained versions of the RBD::mClover transgene show similar expression patterns and obvious localization to vesicles. Scale bars in DIC panels indicate 5 μm.

When characterized by SDS-PAGE followed by Western Blotting using anti-GFP antibodies to detect the mClover tag, we found cell pellets of the secreted and ER retained versions of the RBD::mClover protein generated a band at the expected molecular weight of ~51 kDa. Similarly, ammonium sulfate precipitated protein from the media of the RBD::mClover secreted construct had a detectable band at ~51 kDa. Of the few recoverable Chloroplast-directed RBD::mClover transformants, all showed a smaller anti-GFP immunoreactive product at ~45 kDa, clearly smaller than the expected 51 kDa product (Fig 2A). Further characterization using anti-SARS-CoV-2 RBD polyclonal antibodies to probe the Western Blots, revealed that while the secreted and ER-Golgi retained proteins were detected as expected 51 kDa bands, the chloroplast directed proteins at ~45 kDa were not detected, despite generating comparatively stronger anti-GFP signal (Fig 2B). It should be noted that a weak cross-reactive band at ~51 kDa is observed in the wild-type algae lysate when blots are probed with the anti-RBD Rabbit Polyclonal antibody. When equal amounts of total cell lysate protein were loaded, darker bands were observed in lysates from the ER-Golgi retained and secreted RBD::mClover strains at the expected molecular weight compared to either the wild type or Chloroplast directed strain (S1 Fig for loading control verification using anti-AtpB).

The chloroplast localized RBD::mClover protein was then purified using anion exchange chromatography followed by anti-GFP magnetic bead immunoprecipitation, and the partially purified protein products were characterized using protein mass spectrometry analysis. The most N-terminal peptide fragment detectable by mass spectrometry in the chloroplast-directed RBD::mClover corresponded to a protein product that would be 9 kDa smaller than the predicted 51 kDa full length mature protein, while a parallel experiment using the ER-Golgi retained protein identified peptide matches across the entire length of the protein (S2 Fig). Additionally, multiple mass spectrometry peptide matches cover the entirety of the C-terminal mClover domain of the Chloroplast-directed version further suggesting the truncation is in the N-terminal region. We PCR-amplified and sequenced the chloroplast localized and ER-Golgi retained versions of the transgenes in the algal transformants to confirm that the truncation of the chloroplast-localized RBD::mClover was not due to a mutation in the integrated nuclear transgene. In both cases, a perfect match to the designed reference sequence was found throughout the RBD::mClover coding region.

To further help down-select and characterize our recombinant RBD constructs, we conducted fluorescence microscopy on live mounted cells. Obvious subcellular localization of mClover fluorescence to the vesicles and the ER-Golgi system is observed in both our secreted and ER-Golgi retained recombinant constructs (Fig 2C–2R). Comparatively, the chloroplast-directed version displays diffuse fluorescence throughout the cell and little co-localization with the Chlorophyll auto-fluorescence of the chloroplast itself (Fig 2G–2J). Collectively, the mis-localization and truncation of the chlorophyll-directed version of recombinant RBD::mClover led us to down-select against this version for further characterization.

### Purification and characterization of the RBD::mClover fusion proteins

**Fig 3:**
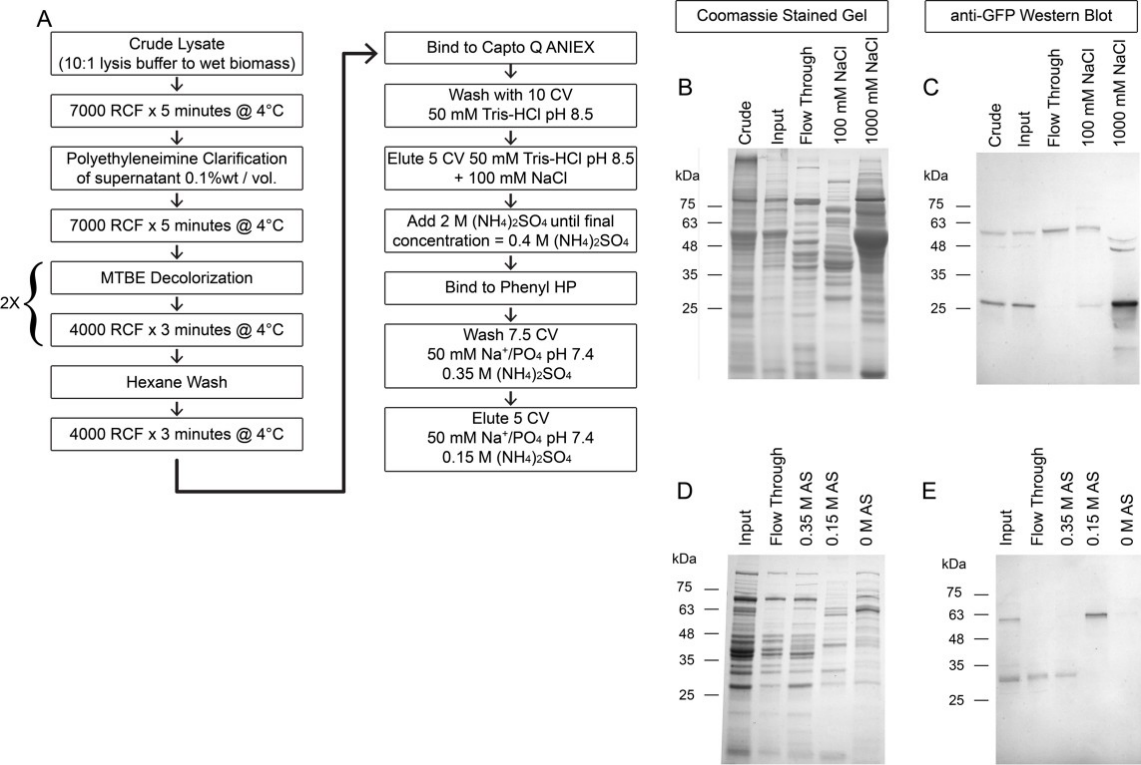
Summary of purification scheme for RBD::mClover retained in the ER-Golgi system. (A) Schematic summarizing our clarification and decolorization protocol prior to column chromatography purification of the RBD::mClover domain through successive Anion Exchange and Hydrophobic interaction resin chemistries. (B) Coomassie stained gel of crude lysate total lysate, Polyethyleneimine/Organic solvent clarified input fraction followed by the Capto Q chromatography Flow through, 100 mM NaCl elution of the RBD::mClover, and 1 M NaCl stripping of the column. Crude and input represent 10 μL of lysate while other fractions represent the same volume of a 20X concentration from the chromatography eluted fractions. (C) Western Blot characterization of the same samples as in panel B but using anti-GFP antibody to probe for the RBD::mClover complex. (D) Coomassie stained gel characterizing the Hydrophobic-interaction purification of the anion-exchange 100 mM NaCl fraction. All samples represent 10 μL of a 20X concentration of each fraction. The input represents the 100 mM NaCl anion exchange fraction after the addition of Ammonium Sulfate (AS) to 0.4M. (E) Western Blot Characterization of the same samples using anti-GFP to detect the RBD::mClover gene product. The RBD::mClover can be separated from the lower molecular weight mClover degradation product.

To characterize the RBD protein produced using *C*. *reinhardtii*, we opted to purify the RBD::mClover from the ER-retained version rather the secreted version, because we noticed that the protein had similar molecular mass, suggesting similar post-translational modifications. Additionally high molecular weight aggregates and low molecular weight degradation products were observed in the precipitated secreted proteins; thus, making the ER-retained version the most optimal option of our three constructs (Fig 2B). Using a variety of chromatography resins (Fig 3), we were able to purify sufficient amount of ER-retained RBD::mClover to characterize the protein for receptor-interaction activity. We found that total RBD::mClover protein was 2.6% of total protein after this partial purification with a starting concentration of ~0.1% total soluble protein using our extraction method. We determined initial recombinant protein yields of 31 μg/gram of total wet biomass and, after partial purification, 1.8 μg/gram of total wet biomass.

### Functional characterization of algae-produced SARS-CoV-2 spike RBD protein

**Fig 4:**
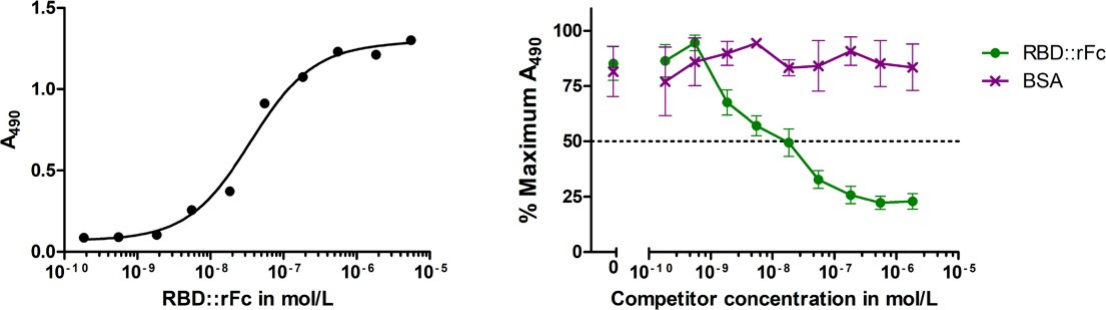
Characterization of algae-produced RBD::mClover through ACE2 receptor binding assays. (A) HEK-cell produced RBD fragment fused to Rabbit IgG Fc fragment (RBD::rFc) binding to immobilized biotinylated recombinant human ACE2 was detected by anti-Rabbit IgG-HRP antibodies and TMB chromogenic reaction. Data shown represent values from one experiment. (B) ACE2 Receptor binding competition assay between a constant concentration of partially purified ER-Golgi Retained Algae-Produced RBD::mClover (~40 nM) and increasing amounts of RBD::rFc or Bovine Serum Albumin showing specific competition. RBD::mClover binding was detected using anti-GFP HRP antibodies. Data points represent mean and error bars represent Standard Error of the Mean of normalized A_490_ signal values over three independent experimental repeats.

The SARS-CoV-2 spike RBD interacts with human host cells through ACE2 receptor binding [4, 6, 29]. To determine if the algae-expressed ER-retained RBD::mClover was correctly folded and functional, we established a human ACE2 receptor binding assay for SARS-CoV-2 RBD-containing proteins. HEK cell line expressed biotinylated soluble human ACE2 receptor was immobilized on Strepavidin coated microtiter plates and used as a substrate for SARS-CoV-2 RBD binding. To validate the assay we first determined that the HEK cell expressed SARS-CoV-2 RBD C-terminally fused to rabbit IgG Fc (RBD::rFc) could bind to the immobilized ACE2, and could identify that the recombinant RBD bound the ACE2 protein at an EC_50_ of ~36 nM (Fig 4A). This affinity is within the nM range previously reported for SARS-CoV-2 RBD-ACE2 interactions [34, 35]. Next we added a constant amount of our partially purified algae-expressed RBD::mClover (40 nM) to each ACE2 receptor coated well, and then titrated in RBD::rFc as a competitor. Using anti-GFP antibodies to detect RBD::mClover bound to the immobilized ACE2 receptor, we find that the RBD::rFc will compete RBD::mClover binding at the ACE2 receptor with an IC_50_ of ~20 nM. Since a roughly equal concentration of HEK cell-produced RBD::rFc, compared to the RBD::mClover, is required to generate ~50% competition at the ACE2 receptor, we suggest that this indicates that the algae-produced RBD displays a similar affinity for the receptor as the mammalian produced version. Further, this competition appears to be specific because addition of Bovine Serum Albumin as a potential competitor does not decrease apparent RBD::mClover binding (Fig 4B).

## Discussion

Here we have demonstrated that production of a correctly folded and functional SARS-CoV-2 spike protein Receptor Binding Domain is possible in the green alga *C*. *reinhardtii*. By fusing the viral protein to a fluorescent mClover protein, we could use a high throughput fluorescent screening strategy to rapidly identify strains of algae expressing sufficient quantities of SARS-CoV2 RBD to test protein accumulation and function. We showed that stably integrated nuclear encoded transgenes, directed to either the ER or secreted from the cell, produce a fusion protein of the expected size that appeared to be correctly folded and post-translationally modified, allowing the protein to specifically bind the ACE2 receptor. Using partially purified ER localized algae SARS-CoV-2 RBD to conduct ACE2 receptor binding interaction assays, we demonstrated that the algae-produced ER-retained version of RBD::mClover did indeed interact in a specific manner with its cognate human host receptor, at a perceived affinity similar to mammalian cell line expressed SARS-CoV-2 RBD, suggesting that the folding and post-translational modifications of the recombinant algae RBD are sufficient to mediate appropriate RBD binding activity.

Although seeming to be expressed at higher levels, the RBD protein targeted to the chloroplast appeared to be truncated. We reason that the truncation is occurring at the N-terminus because a loss of ~5 kDa from the C-terminus would eliminate multiple beta-sheets from the mClover beta-barrel thus abrogating fluorescence; yet, we can readily detect mClover fluorescence under the microscope and using a microtiter plate reader. Lack of mass spectrometry peptide matches in the N-terminus of the chloroplast-directed version but strong coverage of the mClover C-terminal supports this. The truncation itself may be occurring before chloroplast translocation because the mClover fluorescence observed under the microscope did not localize well with the chlorophyll auto-fluorescence.

Collectively these data demonstrate the suitability of algae as a platform for the rapid production of recombinant proteins that are correctly folded and post-translationally modified to create a functional protein. Because algae can be grown at scale for a fraction of the cost of mammalian cell lines, this system offers the potential to produce this recombinant viral protein for a variety of uses including as an antigen to detect serum antibodies against SARS-CoV-2 RBD or even as a potential viral antigen for vaccine development, in a rapid and cost-effective manner.

Recently SARS-CoV-2 RBD has been reported to be produced in other photosynthetic systems. SARS-CoV-2 RBD has been transiently expressed in *N*. *benthamiana* by agroinfiltration of *Agrobacterium* carrying recombinant expression vectors with a reported yield of 8μg/gram leaf mass of highly purified recombinant RBD able to bind to the ACE2 receptor (compared to our 31 μg/gram wet biomass of unpurified RBD and 1.8 μg/gram wet biomass of partially purified RBD). Of note, the *N*. *benthamiana* RBD expression construct used a C-terminal 8xHis tag to enable Ni–NTA affinity resin purification from relatively crude leaf extract [20]. A similar His-tagged transgene design could be adopted to facilitate more efficient and rapid purification of RBD and other recombinant proteins from *C*. *reinhardtii* since protein purification from land plants and algae face similar problems of heavy cell wall debris and pigment loads that make many conventional purification workflows developed for bacteria, yeast, or mammalian cells often untenable. In the current study, we needed to develop a novel de-fatting/decolorization and clarification method to avoid irreversible fouling of our protein chromatography columns and loss of resolution during chromatographic separation.

Transient expression of SARS-CoV-2 RBD in both *C*. *reinhardtii* and *C*. *vulgaris* at 1–2 μg/gram total wet biomass was recently reported using an *Agrobacterium*-mediated transformation method and vectors adapted directly from *N*. *benthamiana* [28]. In these transiently transformed algae, receptor binding activity was not assessed, nor was the long term expression levels of the protein beyond 72 hrs post-transformation characterized. Comparatively our expression method involves nuclear genome integration with transformants that have stably expressed the RBD transgene without obvious attenuation for approximately one year at the time of this publication. Moreover, this nuclear transformation method generates an order of magnitude higher expression level of RBD::mClover per gram of wet biomass in *C*. *reinhardtii* and only takes three weeks from transformation to initial transformant characterization by Western Blotting. The higher expression level likely stems from the combined use of initial Zeocin antibiotic resistance selection (*i*.*e*. the resistance gene stoichiometrically sequesters rather than degrades Zeocin) followed by a fluorescence-based down-selection method for primary nuclear genome transformants as well as the use of endogenous promoters, untranslated regions, and a codon-optimized RBD sequence. Use of stronger endogenous or synthetic promoters [36] in new transgene designs as well as the addition of more introns [37] in to the RBD-coding sequence could be used to boost recombinant RBD expression from the nuclear genome in future optimized expression constructs. Further improvements on recombinant RBD expression can be made through mating and mutagenesis followed by high throughput selection methods such as flow cytometry since our RBD transgene contains a fluorescent protein tag. Previously, our group demonstrated a 15-fold improvement in a chloroplast encoded transgene through such an approach over just a few months of crossing, mutagenesis, and selection [33]. Transformation of the *C*. *reinhardtii* chloroplast genome could be attempted as an alternative strategy since markedly higher recombinant transgene expression levels have been reported from constructs integrated in to the plastid genome [38, 39]. However, it should be noted, that many of the chloroplast genome engineering approaches in *C*. *reinhardtii* that generate the highest transgene expression levels also attenuate or eliminate the photosynthetic ability of the organism, thus removing the option of low cost photosynthetic cultivation [38, 40].

To enable large scale photosynthetic production of recombinant proteins and to drive down production costs, reliable long-term expression from well characterized strains will be required to avoid both batch variation and the need to constantly re-transform the system. While transient expression of SARS-CoV-2 antigens may be more facile in *N*. *benthamiana*, stable nuclear genome integration and overall productivity in *C*. *reinhardtii* or closely related species may be superior if we consider daily productivity of algal systems under photosynthetic conditions compared to land plants. When projecting commercial scale production of microalgae in large open ponds, typical productivities range from 40–80 Mg/ha/yr at a cost of approximately $2,000/Mg whole cell dry biomass [41–43]. In comparison, tobacco plants typically yield 2.5–5 Mg/ha/yr [44], potentially resulting in more than a ten-fold increase in biomass productivity when using microalgae as a production platform. It has also been shown that *C*. *reinhardtii* is capable of being grown using industrial fermentation processes, producing approximately 200 mg/L/hr of biomass [22, 45]. Assuming that the nuclear encoded transgene design can be further improved and coupled with breeding to generate an expression strain generating 1% total soluble protein as recombinant RBD, upwards of 400 kg/ha/yr of RBD at annual production cost of $80,000 could be achieved. This excludes the cost of downstream biomass processing and purification. With this considered, other expression systems currently display more facile protein purification than green algae does and further improvements will be required to fully realize the potential of green algae as a recombinant protein production platform.

In light of multiple new SARS-CoV-2 variants containing mutations in the spike RBD that correlate with the strains being more transmissible and/or displaying immune system evasion [46, 47], developing rapidly engineered and scalable expression systems will remain vital despite the roll out of vaccines. For example, serology assays that specifically detect immunity to new variants or booster vaccines against such variants will be key tools as the COVID19 pandemic stretches in to the 2020s. To this end, it would be interesting to apply the rapid but transient agrobacterium mediated transformation approaches to rapidly advance through design-build-test cycles on multiple variations of RBD transgene constructs, such as testing variations of affinity tags or additional subcellular localization approaches, and then apply those lessons to stable integration methods for scale-up.

## Materials and methods

### Strain and culture conditions

*Chlamydomonas reinhardtii* strain CC124 was used throughout experiments. Cells were grown on standard TAP media [48] under 24-hr light conditions at ~22–25°C at a photon flux of 125 μE/m^2^/sec on shaker tables rotating at 110 RPM.

### SARS-CoV2 RBD sequence design and optimization

The genomic sequence of SARS-CoV-2 (Wuhan-Hu-1 strain) was retrieved from the NCBI (NC_045512). Amino acids 319 to 542 of the spike protein comprising the RBD were codon optimized for *C*. *reinhardtii* nuclear genes [49]. Briefly, we generated a codon-usage table using the mRNA sequences derived from the top 1600 (~10%) nuclear genes expressed in *C*. *reinhardtii* in TAP media under light growth conditions [50]. Codon optimization was then performed using Unipro UGENE [51] with final codon rotations to avoid direct repeats or regions of >70% GC content that were flagged as difficult to synthesize by the IDT gBlock design tool. The full codon optimized sequence was synthetized as a double-stranded gBlock oligonucleotide by IDT (Integrated DNA Technologies Coralville, IA).

### Expression vector design and assembly

The expression vector was derived from previously published pBR9 [26], pOPT mClover [31] and pHyg3 [32]. Standard PCR-based amplification to add overlap-adaptors and Gibson-style assembly methods (HiFi Assembly Kit, New England Biolabs, Ipswich, Massachusetts) were used to assemble the final vectors. The entirety of the *C*. *reinhardtii-*specific payload was sequenced by Sanger Method at Eton Bioscience (San Diego, CA).

After examining available structures of the SARS-CoV-2 S- protein, a slightly smaller version of the RBD, comprising amino acids 319–537, was chosen for further subcloning to avoid a cysteine that is normally in a disulfide bridge with the “stalk” of the S-protein and may pose potential stability or intermolecular dimerization issues. Three different sub-cellular localization strategies were chosen for RBD expression. First, a chloroplast-localized version was produced by N-terminal fusion of a previously characterized psaE chloroplast transit sequence localization signal [52] to the RBD domain generating vector pRMC1. The genomic sequence spanning the psaE start codon until four amino-acids after the CTS cleavage site were amplified from *C*. *reinhardtii* strain CC125 genomic DNA purified by standard Phenol/Chloroform/SDS lysis and Isopropanol/Ethanol precipitation method. The second construct contained a secretion peptide motif (as predicted by SignalP5.0) from the Pherophorin 2 gene (PHC2) [53]. The signal peptide was specifically chosen because PHC2 is known to be highly abundant in the *C*. *reinhardtii* secretome [54]. The genomic locus was amplified and placed upstream of the RBD coding sequence, as indicated above for the psaE CTS, to generate vector pRMC2. Finally, an ER-Golgi retained version of the RBD was generated by adding an additional KDEL retention motif to the C-terminus of the RBD coding sequence in pRMC2 to generate pRMC3. All vector maps are included as GenBank files in S2 File.

### Algae transformation

All plasmid vectors were prepared by Qiagen midi prep and 10–20 ug of DNA linearized by digest with KpnI-HF and XbaI restriction enzymes at 37°C in NEB Cutsmart buffer with enough units of activity to generate ~5X overdigest in two hours (New England Biolabs, Ipswich, MA). Reaction was stopped and DNA partially purified by NaCl/Isopropanol precipitation followed by three 70% Ethanol washes and resuspension in 1 mM Tris-HCl, 0.1 mM EDTA overnight at 4°C.

A starter culture of *C*. *reinhardti* strain CC124 was grown in standard TAP media to near saturation (~3x10^6^ cells/mL) then diluted back in to 0.25x10^6^ cells/mL in 300 mL of TAP media in a baffled flask ~24 hours before transformation. The next day, cells were collected at a density of 0.75–1.0x10^6^ cells/mL by centrifugation at 3000 rcf x 10 minutes at 16°C in sterile disposable conical bottom centrifuge bottles. The cells were then resuspended in GeneArt® MAX Efficiency® Transformation Reagent for Algae (Thermo Scientific, Waltham, MA) and processed according to manufacturer’s instructions. Each construct was electroporated using a Gene Pulser Xcell Electroporation System (Bio-Rad Laboratories, Hercules, CA) with 2 ug of linearized vector DNA. After over-night recovery in 40 mM Sucrose in TAP media, cells were pelleted, resuspended in 5 mL of fresh TAP media and 200 uL of cells were spread on 10 cm diameter plates containing 15 μg/mL Zeocin and 30 μg/mL Hygromycin B (Thermo Scientific, Waltham, MA) in TAP+15 g/L agar media.

### Transformant down-selection

Ten days after electroporation, individual colonies were transferred into 96-well microtiter plates containing liquid TAP media; CC124 strain and previously generated GFP-expressing strain [33] were included in each plate as internal positive and negative controls. mClover fluorescence, chlorophyll fluorescence, and optical density were quantified following previously published methods [33]; mClover fluorescence was normalized to chlorophyll to account for differences in cell density. The seven highest normalized mClover fluorescence clones were then inoculated into 50 mL shaker cultures, and grown for three days until late log phase was achieved (1-3x10^6^ cells/mL), at which point one mL of culture was harvested for further analysis. For pRMC1 and pRMC3 the supernatant was aspirated off and the pellet was snap frozen at -70°C. For pRMC2 the 50 mL of supernatant was recovered, centrifuged a second time to remove any residual cell debris, and then 40 mL of culture media snap frozen. Subsequently, secreted RBD::mClover was precipitated by addition of solid ammonium sulfate to a final concentration of 302 g/L (~50% saturation at 0°C), followed by incubation over night at 4°C with mixing, then centrifugation at 16x10^3^ rcf. The supernatant was removed and the pellet was resuspended in 200 μL of 50 mM Tris-HCl pH 8.5. In preparation for Western Blot analysis, cell pellets were quick thawed in a room temperature water bath, lysed in 100 μL of Bugbuster (Sigma Aldrich, St. Louis, Missouri) for 10 minutes on ice with the addition of Basemuncher (Abcam, Cambridge, MA), cell debris was pelleted (5 minutes x 16x10^3^ rcf at 4°C) and 60 μL of clarified supernatant recovered. Proteins were denatured by addition of 20 μL 4X Laemmli buffer with 10% BME and then heat treated at 80°C for 10 minutes before 20 μL were loaded on to a Tris-Glycine TGX 12% acrylamide gel for SDS-PAGE (Bio-Rad Laboratories).

### Western Blotting and Coomassie staining

After separation on acrylamide gels, proteins were transferred to nitrocellulose membranes via semi-dry transfer (15 V x 60 minutes). Membranes were washed 3x2 minutes with TBSMT (50 mM TrisHCl pH 7.4, 150 mM NaCl, 0.05% Tween-20) then blocked for 20 minutes in Haycock’s blocking solution; 1% wt/vol Polyvinylpyrrolidone in TBSMT [55]. Recombinant gene product was either detected with Goat anti-GFP conjugated to Alkaline Phosphatase (ab6661 Abcam, Cambridge, Massachusetts) at 1:5000 dilution or 1:5000 Rabbit Polyclonal anti-SARS-CoV2 RBD (Cat# 40592-T62 Sino Biological, Chesterbrook, PA), in Haycock blocking solution. The latter was then detected with Goat anti-Rabbit::AP at 1:10,000 dilution. All antibodies were incubated for 60 minutes at room temperature and 4x3 minute washes in TBSMT were conducted between steps. Chromogenic development was performed by BCIP/NBT reaction in Alkaline Phosphatase buffer (100 mM TrisHCl pH 9.5, 5 mM MgSO_4_, 0.01% Tween-20) before quenching the reaction by washing in distilled water followed by TBSMT. For anti-AtpB loading controls in S1 Fig, the above protocol was followed but Rabbit anti-AtpB (Beta subunit of ATP synthase; chloroplastic and mitochondrial; Agrisera, Sweden) was used as the primary antibody at 1:20,000 dilution. For all Coomassie stained gels, proteins were separated by SDS-PAGE as above and then stained with SimplyBlue SafeStain (Thermo Fisher Scientific) as per manufacturer’s standard protocol.

### Protein quantification

Soluble protein was calculated using the Pierce Coomassie (Bradford) Protein Assay Kit (Thermo Fisher Scientific) according to the manufacturer’s instructions after lysis with the Bugbuster lysis cocktail. For total soluble protein from lysate, to control for potential confounding detergent effects, an equivalent amount of Bugbuster cocktail was added to the BSA standards. RBD::mClover quantification was done through semi-quantitative Western Blotting using HEK-cell produced RBD::6xHis (Cat# 40592-V08H Sino Biological) as a standard at half-log dilutions from 100 μg/mL to 10 ng/mL. Pseudo-densitometry was then performed using Fiji [56] and the standard curve and linear regression generated in Graphpad Prism 5.0 (GraphPad Software, San Diego, CA). Total soluble protein from lysate and purified protein values are presented as calculations from one sample.

### Genotyping

The strains verified above as expressing RBD::mClover were subject to genomic DNA extraction using the Chelex 100 boil method [57]. Primers flanking the transgene (FDX1_seq1 5’-TAGCGCAGCTTCGCCTACAT-3’ and BTpro_seq2 5’-AGCTCGAGTGGCCTGTGTAGA-3’) were then used to amplify the entirety of the RBD::mClover coding region using Q5 DNA polymerase (New England Biolabs). PCR amplicons were size-verified by electrophoresis on a 0.8% TAE agarose gel stained with SYBR Safe (Thermo Scientific, Waltham, MA) and then excised and purified using the Wizard® SV Gel and PCR Clean-Up System kit (Promega, Madison, WI) according to manufacturer’s protocol. The purified amplicons were then subcloned in to pJet1.2 using the CloneJET PCR Cloning Kit (Thermo Scientific, Waltham, MA) according to manufacturer’s protocol and the ligation product transformed in to DH5alpha chemically competent cells (New England Biolabs), miniprepped, and sequenced using primers AJB5_seq1, RBD_seq1, RBD_seq2 and Clov_seq1 (5’-GCAGACCCTGAACTTCG-3’, 5’-CCGGCAAGATCGCTGACTAC-3’, 5’-GATTGGACTTGCGGAACAGG-3’, and 5’-GCTGAACTTGTGGCCGTTC-3’, respectively) as well as the kit provided pJet1.2F/R primer pair.

### Microscopy

Cells were grown in liquid TAP media without antibiotics to mid-log phase (~1x10^6^ cells/mL) on a rotary shaker. Cells were live mounted on conventional microscope slides in TAP media with glass coverslips. Images were captured on a Delta Vision (Applied Precision Inc., Issaquah, WA) optical sectioning microscope system composed of an Olympus IX71 inverted microscope (Center Valley, PA) equipped with an Olympus UPlanSApo 100×/1.40 objective and a CoolSNAP HQ2/ICX285 camera (Photometrics, Tucson, AZ). The Tetramethylrhodamine isothiocyanate (TRITC) filter (EX555/28 and EM617/73) was used to image Chlorophyll auto-fluorescence while the GFP filter (EX470/40 and EM525/36) was used to image mClover fluorescence. Image acquisition was performed using Resolve3D SoftWoRx-Acquire (Version 5.5.1, Applied Precision Inc, Issaquah, WA). Brightness and contrast were adjusted identically across all images using FIJI software.

### Scaled cultivation and bulk protein purification

Algae cultures were grown in a semi-continuous fashion in 1 L baffled flasks in TAP media wherein 95% of the culture was harvested via centrifuge and replaced with new media every two days; harvested call pellets were snap frozen until lysis. Cells were lysed in 1X Bugbuster (diluted from 10X buffer free stock) buffered with 50 mM Tris-HCl pH8.5 containing 1X Pierce Protease Inhibitor Cocktail (Cat#A32955; Thermo Scientific, Waltham, MA), 250 U/mL of Basemuncher endonuclease (ab270049, Abcam, Cambridge, MA), 1 mM DTT, 1 mM EDTA. 10 mL of lysis buffer was added to each gram of snap frozen wet biomass with typically 3–6 grams of wet biomass being processed at once. To the partially clarified cell lysate 10% wt/vol Polyethylenimine (Cat#408719, Sigma-Aldrich, St. Louis, MO), adjusted to pH 8.5 by addition of concentrated HCl was added to a final concentration 0.1% wt/vol. The lysate was then centrifuged at 6000 rcf at 4°C for 5 minutes and supernatant recovered. The lysate was then further decolorized by gently shaking against an equal volume of Methyl tert-Butyl Ether (MTBE) twice and then Hexanes (Fisher Scientific). The aqueous layer was then recovered, filtered through a 0.45 μm PES syringe filter and applied to a Capto Q anion exchange resin for column chromatography purification on an Akta pure 150 system fitted with an external sample pump (Cytiva, Amersham, UK).

### Protein chromatography

A 5 mL HiTrap Capto Q resin anion exchange column (Cytiva) was equilibrated with 5 column volumes (CV) of 50 mM TrisHCl pH8.5. The clarified and decolorized lysate was then applied to the column through the system pump at 3 mL/minute. The column was washed with 10 CV equilibration buffer. The RBD::mClover enriched fraction was eluted with 5 CV of 100 mM NaCl in Tris-HCl pH8.5 buffer. During bulk purification ~20 mL of lysate was bound to the column before binding capacity for the RBD was reached. When 40 mL of clarified lysate was processed for bulk purification, 20 mL of lysate was first bound to the column, washed, and eluted as above before the column being immediately stripped by the addition of 5 CV of 1 M NaCl, then 5 CV of 1 M NaOH, before re-equilibrating with 10 CV 50 mM Tris-HCl and performing a second purification cycle. A 5 mL HiTrap Phenyl HP resin prepacked Hydrophobic Interaction column (Cytiva) was equilibrated with 5 column volumes (CV) of 0.4 M (NH_4_)_2_SO_4_, 50 mM Sodium Phosphate buffer pH 7.4. Immediately after Anion Exchange fractionation, the combined 100 mM NaCl fractions containing the RBD::mClover were brought to 0.4 M (NH_4_)_2_SO_4_ by addition from a 2 M stock solution. The RBD::mClover fraction was then applied to the Phenyl HP resin at a rate of 3 ml/min before being washed with 7.5 CV of 0.35 M (NH_4_)_2_SO_4_, 50 mM Sodium Phosphate buffer pH 7.4. The bound RBD::mClover was then eluted by the addition of 5 CV of 0.15 M (NH_4_)_2_SO_4_, 50 mM Sodium Phosphate buffer pH 7.4. The eluted fraction was then concentrated using Amicon Ultra 15 diaconcentrators with a 10kDa cutoff (Cat # UFC901024, Millipore, Temecula, CA) and buffer exchanged 3 times with PBS pH7,4 (Cat # 10010023 Gibco, Waltham, MA) before concentrating down to 3 mL.

### ACE2 receptor binding assay

Strepavidin coated microtiter plates (Cat#15124, Thermo Fisher) were washed 3X in Receptor Assay Blocking buffer (25 mM TrisHCl, 150mM NaCl, pH 7.4, 0.1% wt/vol Bovine Serum Albumin, 0.05% vol/vol Tween-20) and then were coated with 50 ng per well of biotylated human ACE2 produced in HEK cells lines (Cat#10108-H08H-B Sino Biological) dissolved in 100 μL of PBS for one hour at room temperature with gentle shaking on an orbital table. HEK cell-produced recombinant SARS-CoV-2 RBD fused C-terminally to rabbit IgG Fc (RBD::rFc, # 40592-V31H Sino Biological) was used as a specific competitor. A dilution series of 100 to 0.01 μg/mL RBD::rFc was made in PBS containing 2 μg/mL of partially purified algae produced ER-Golgi retained RBD::mClover including a no-RBD::rFc reference. As a negative control, Bovine Serum Albumin (Cat# 23209, Thermofisher) was added at the same molar concentrations as RBD::rFc. Wells were washed four times with Receptor Assay Blocking buffer and then filled with the RBD competitor solution at 30 μL/well for 1 hour at room temperature as above. Wells were washed four times and then filled with 100 μL of Goat anti-GFP conjugated to Horseradish Peroxidase (ab6663, Abcam) diluted 1:3000 in Receptor Assay Blocking buffer. After a 30-minute incubation, wells were washed four times and then 100 μL of Pierce TMB substrate kit added. The chromogenic reaction was incubated at room temperature for 20–25 minutes and then quenched with 100 μL of 2 M HCl. The absorption was then measured at 490 nm using a Tecan Infinite m200 Pro plate reader. Receptor-competition assays were performed as three independent experimental runs. The data are represented as the mean A_490_ signal normalized to the maximum absorption of each experimental run and variance is represented as the Standard Error of the Mean. Initial confirmation of RBD::rFc binding to the ACE2 receptor was performed as above but by simply adding RBD::rFc at increasing concentrations from 0.01 to 300 ug/mL and detected with 1:3000 Goat anti-Rabbit IgG-HRP (Cat#AP307P, Sigma). A single experimental run is represented in Fig 4. In both experiments, data were plotted using Graphpad Prism. For the initial confirmation of RBD:rFC binding, a one-site total binding non-linear regression was used to calculate the EC_50_.

### Characterization by protein mass spectrometry

Protein lysate from the Chloroplast-directed and ER-Golgi Retained strains were prepared as above and RBD protein purified by Anion exchange chromatography using Capto-Q resin. It should be noted that the ER-Golgi retained version of RBD::mClover eluted at 100 mM NaCl in one fraction when a 100 mM stepped gradient from 0 to 500 mM NaCl was tested, while the Chloroplast directed version eluted over a broad range of salt concentrations from 200 to 400 mM NaCl, suggesting marked differences in affinity to the Capto Q anion exchange resin. The RBD::mClover enriched fractions where then diaconcentrated and buffer exchanged three times against 50 mM Tris-HCl, pH 8.5. These samples were then further purified using Anti-GFP mAb-Magnetic Beads (Cat# D153-11, MBL International, Woburn, MA) according to manufacturer’s protocol except for washing with 50 mM Tris-HCl pH 8.5 with 0.05% Tween-20. Samples were submitted for mass spectrometry at the University of California, San Diego’s Biomolecular and Proteomics Mass Spectrometry Facility as either on-bead digestions or as excised bands from SDS-PAGE gels stained with SimplyBlue Coomassie stain according to manufacturer’s instructions (Thermo Scientific).

## Supporting information

S1 Fig Protein Loading control demonstration for cell lysates. Same amounts of total soluble protein lysate (5 μg as determined by Bradford assay) were loaded per well for SDS-PAGE analysis. Chloroplastic AtpB protein was treated as a “house keeping” gene and detected with anit-AtpB antibodies. Approximately equal staining intensity is seen across each lane. These samples are paired with the cell lysate samples in Fig 2.
(TIF)

S2 Fig Summary of protein mass spectrometry data for chloroplast localized and ER-Golgi retained RBD::mClover. (A) Mature peptide sequence of chloroplast directed RBD::mClover (lacking PsaE chloroplast transit signal peptide) (B) Mature peptide sequence of ER-Golgi Retained RBD::mClover (lacking PHC2 secretion signal peptide). Trypsinized peptide fragment matches are highlighted in red. RBD sequence is underlined.
(TIF)

S1 Raw images Raw images for all Western Blots and Coomassie Gels. Title at top of each image corresponds to panel in main text figures.
(PDF)

S1 File Raw micrograph captures at original bit depth in Deltavision file format. File names correspond to panels in Fig 2.
(ZIP)

S2 File Vector maps in GenBank format for pRMC1/2/3. (ZIP)

S1 Data Raw values to recreate graphs in Fig 4. Provided as Excel Spreadsheet with annotations in file.
(XLSX)
