# Supplementary figures and images for: Recombinant production of a functional SARS-CoV-2 spike receptor binding domain in the green algae Chlamydomonas reinhardtii
Source: PLoS One. 2021 Nov 18;16(11):e0257089. doi: 10.1371/journal.pone.0257089 (PMC8601568; doi:10.1371/journal.pone.0257089)

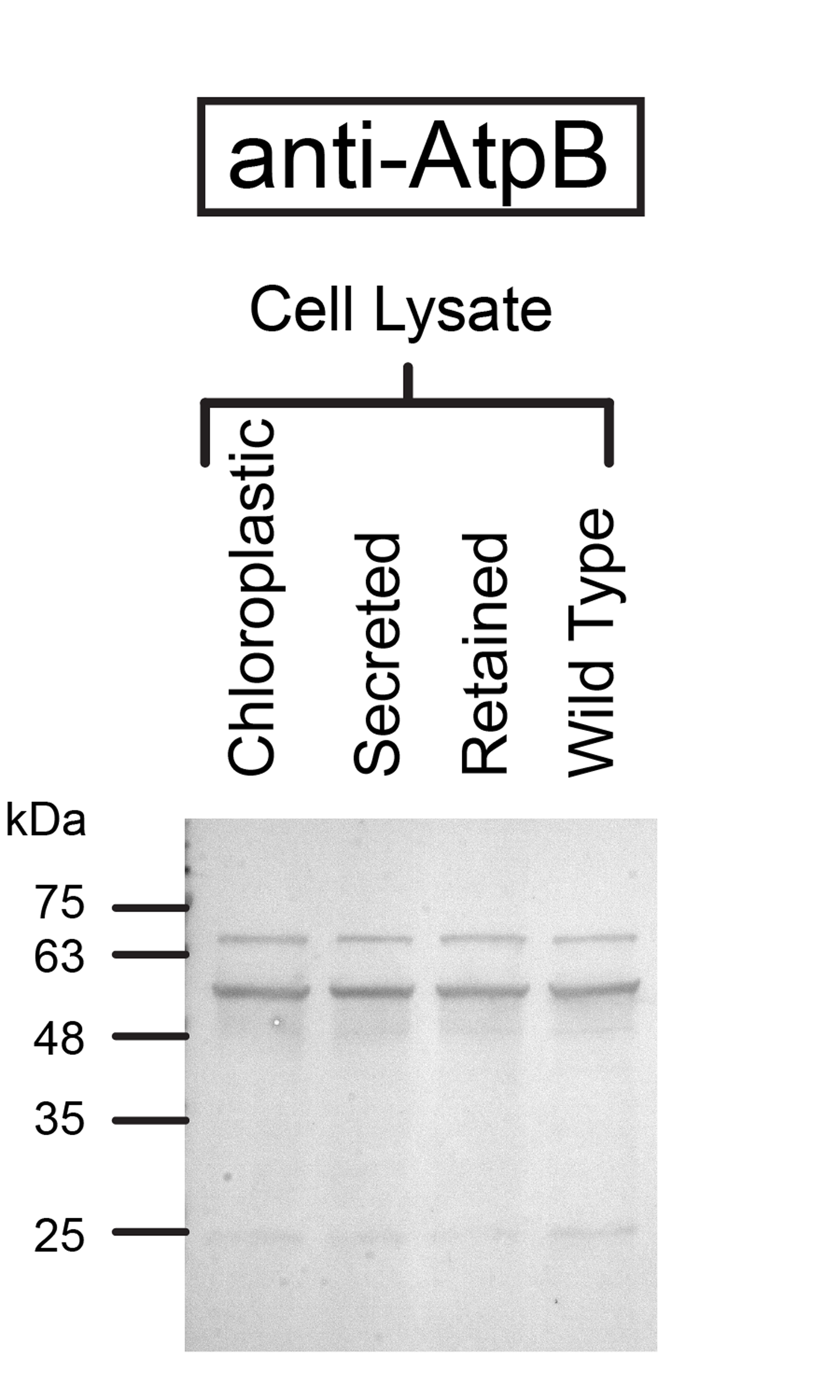

Supplement: S1 Fig — Same amounts of total soluble protein lysate (5 μg as determined by Bradford assay) were loaded per well for SDS-PAGE analysis. Chloroplastic AtpB protein was treated as a “house keeping” gene and detected with anit-AtpB antibodies. Approximately equal staining intensity is seen across each lane. These samples are paired with the cell lysate samples in Fig 2. (TIF) [file pone.0257089.s001.tif]

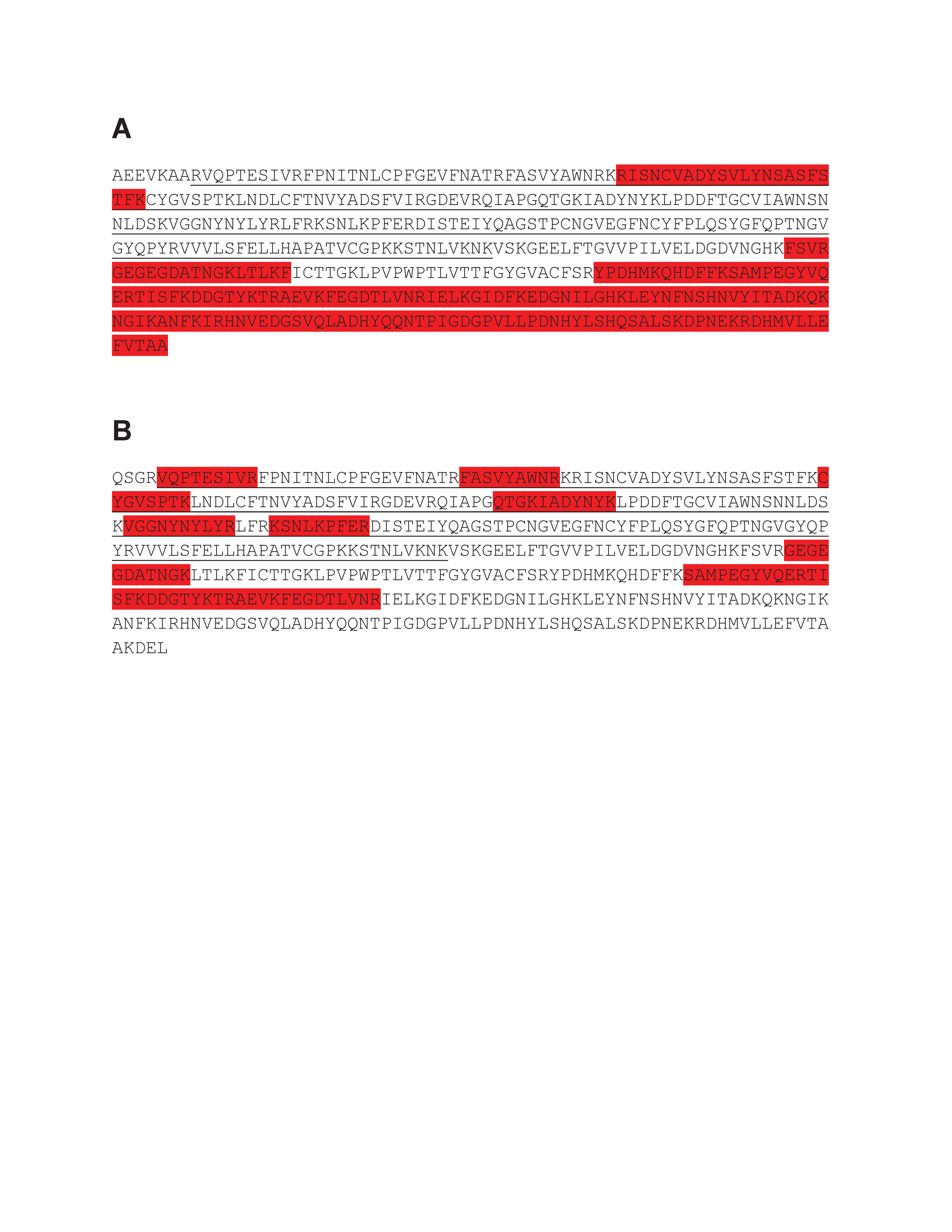

Supplement: S2 Fig — (A) Mature peptide sequence of chloroplast directed RBD::mClover (lacking PsaE chloroplast transit signal peptide) (B) Mature peptide sequence of ER-Golgi Retained RBD::mClover (lacking PHC2 secretion signal peptide). Trypsinized peptide fragment matches are highlighted in red. RBD sequence is underlined. (TIF) [file pone.0257089.s002.tif]

# Figure 2A

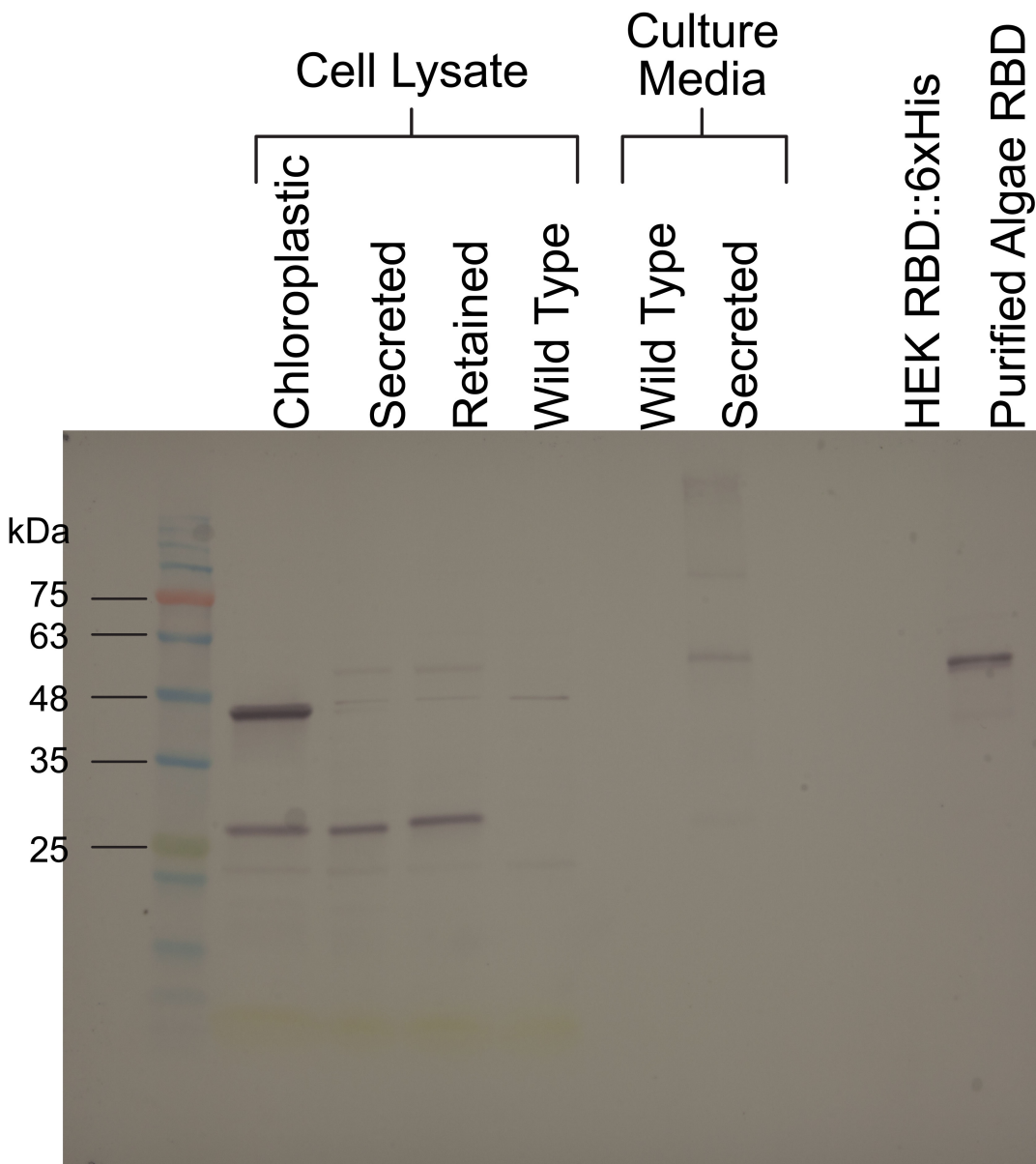

# Figure 2B

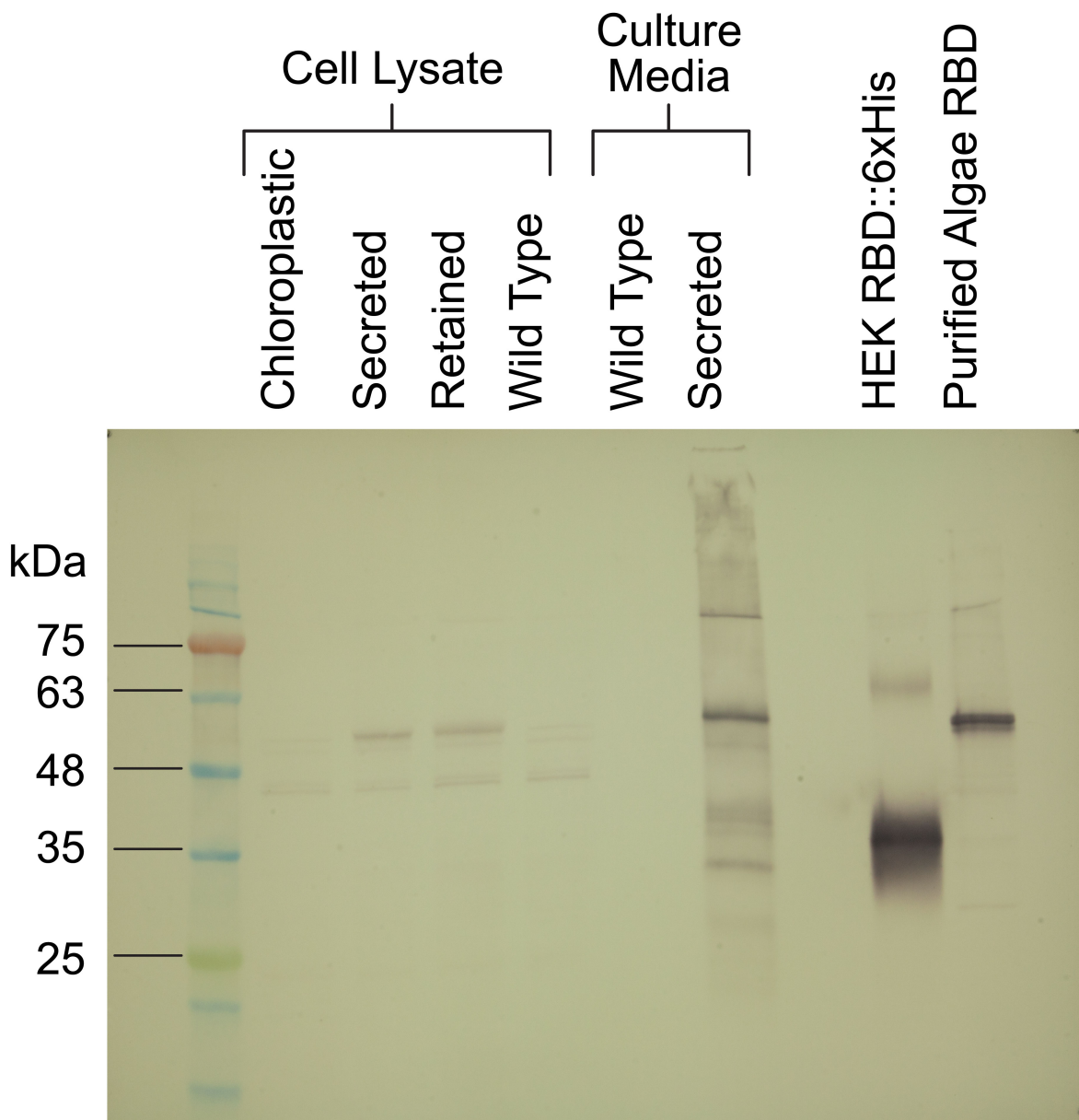

# Figure 3B

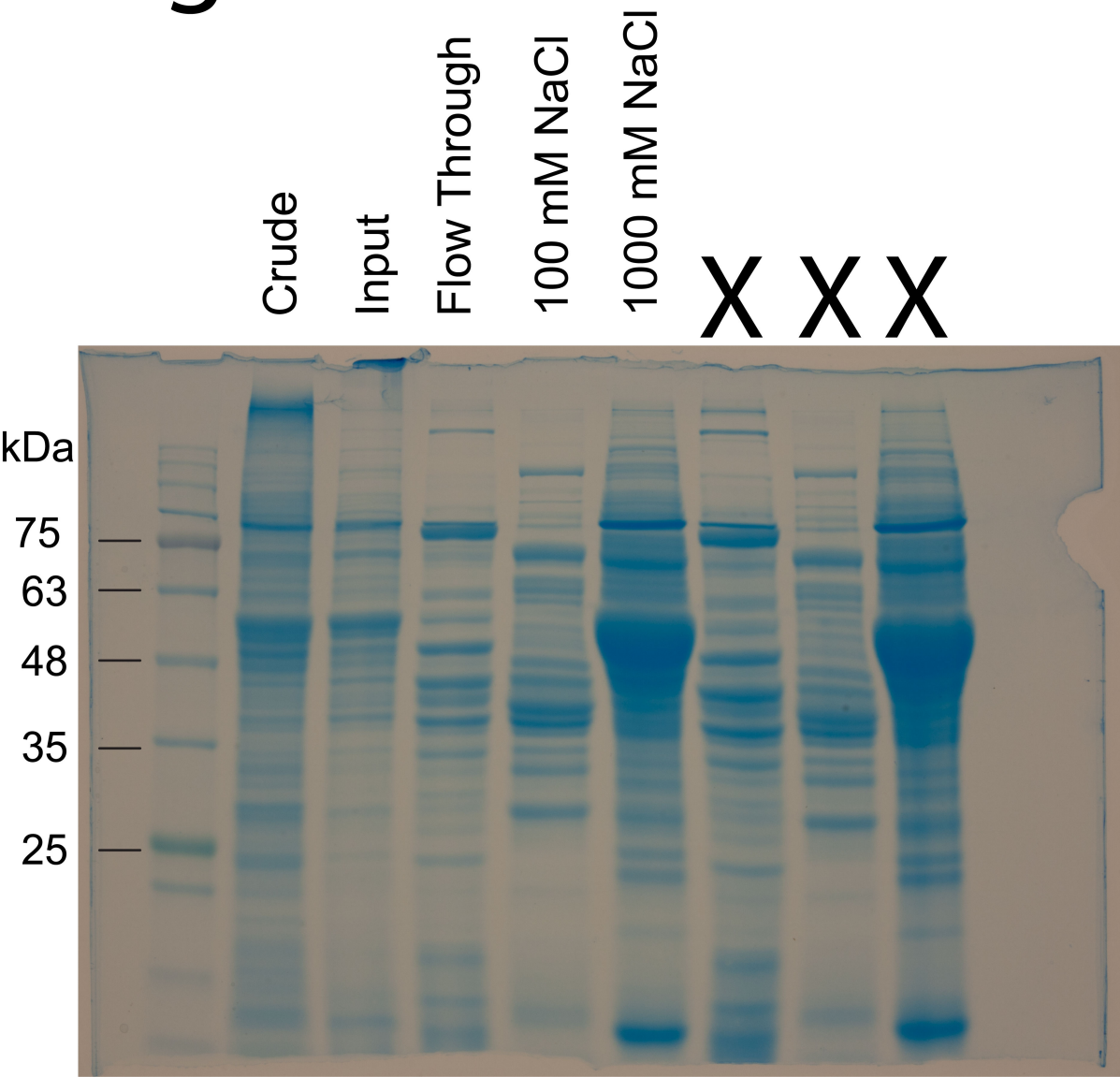

# Figure 3C

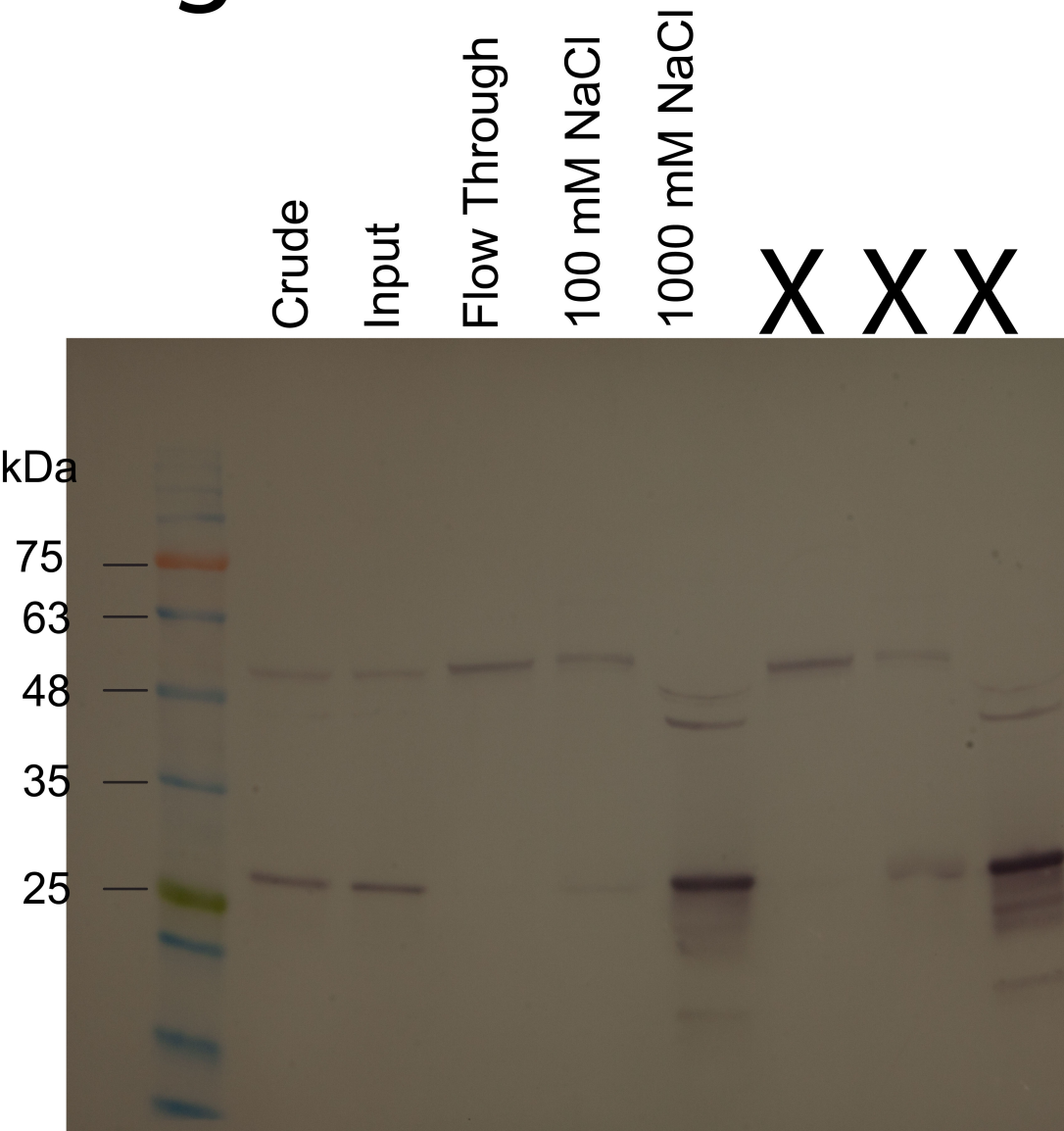

# Figure 3D

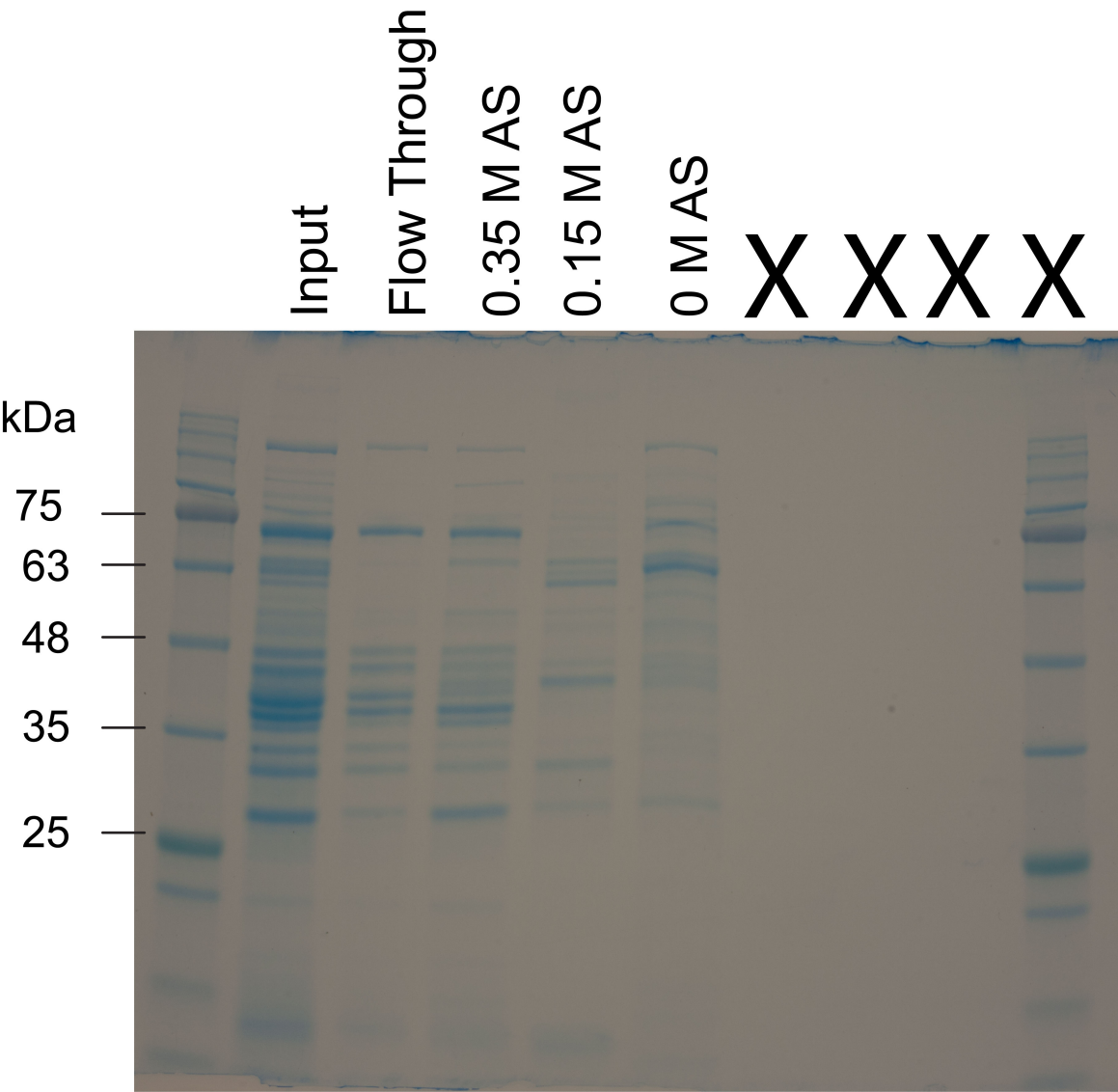

# Figure 3E

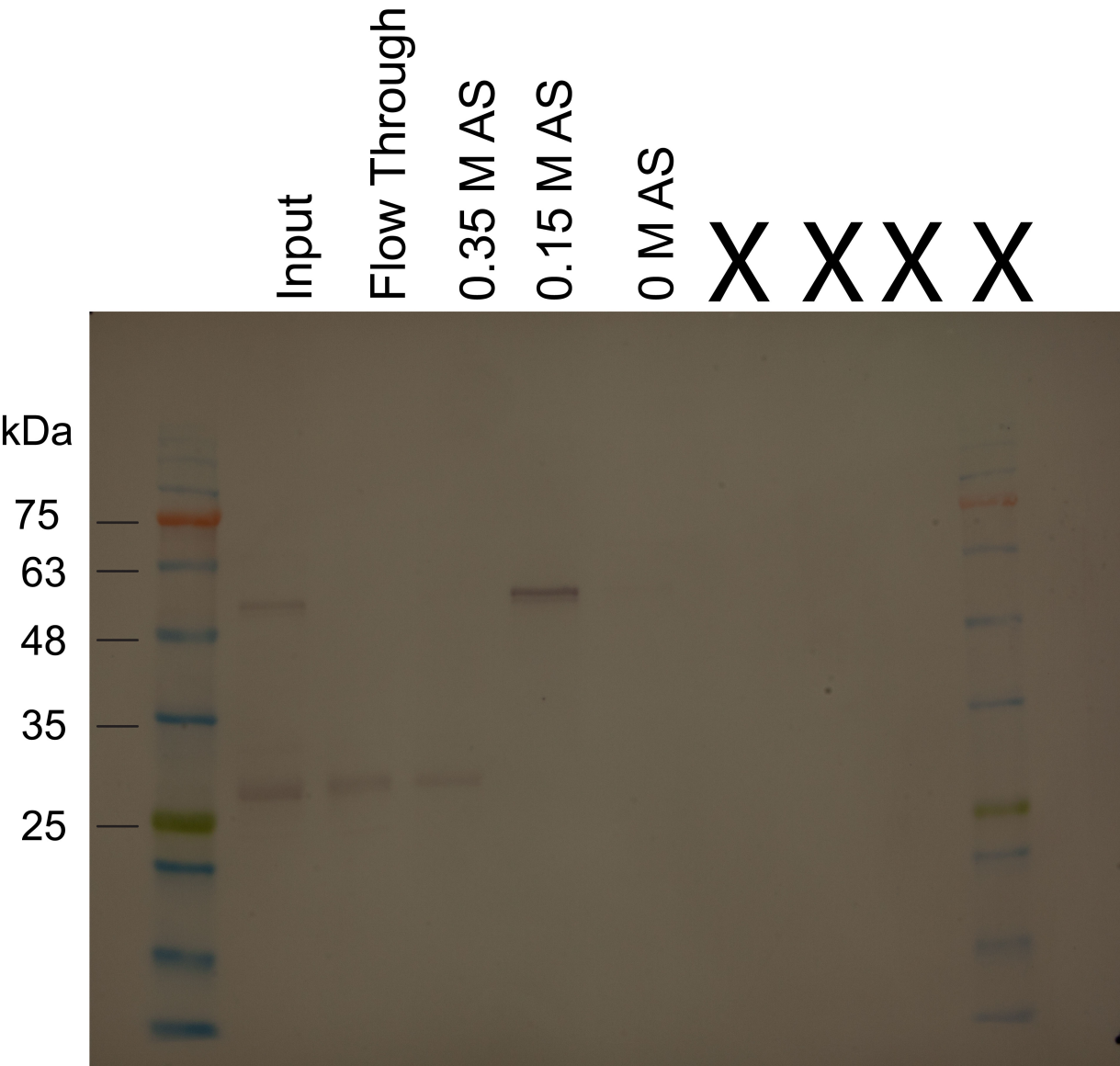

# Figure S1

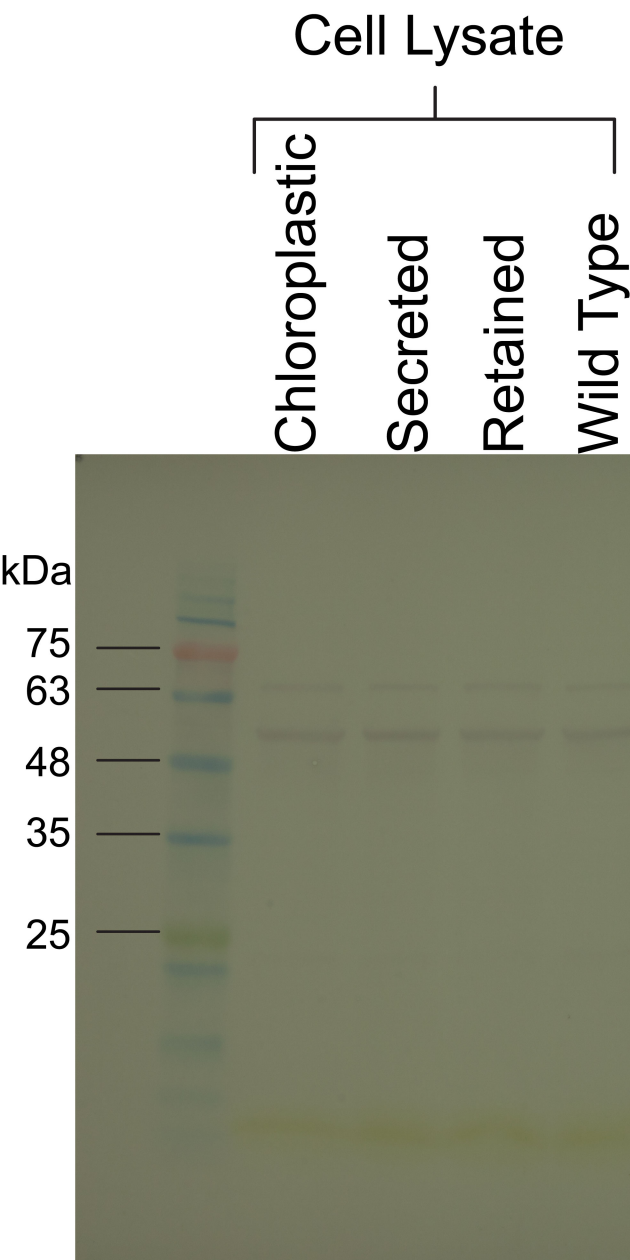

Supplement: S1 Raw images — Title at top of each image corresponds to panel in main text figures. (PDF) [file pone.0257089.s003.pdf]
